# Size and shape heterodonty in the early Permian synapsid *Mesenosaurus efremovi*

**DOI:** 10.1111/joa.14034

**Published:** 2024-03-02

**Authors:** Tea Maho, Sigi Maho, Joseph J. Bevitt, Robert R. Reisz

**Affiliations:** ^1^ Department of Biology University of Toronto Mississauga Mississauga Ontario Canada; ^2^ Dinosaur Evolution Research Center, International Center of Future Science Jilin University Changchun Jilin China; ^3^ Australian Centre for Neutron Scattering Australian Nuclear Science and Technology Organisation Lucas Heights New South Wales Australia

**Keywords:** amniote, computed tomography, dentition, heterodonty, histology, Permian, ziphodonty

## Abstract

Paleozoic synapsids represent the first chapter in the evolution of this large clade that includes mammals. These fascinating terrestrial vertebrates were the first amniotes to successfully adapt to a wide range of feeding strategies, reflected by their varied dental morphologies. Evolution of the marginal dentition on the mammalian side of amniotes is characterized by strong, size and shape heterodonty, with the late Permian therapsids showing heterodonty with the presence of incisiform, caniniform, and multicuspid molariform dentition. Rarity of available specimens has previously prevented detailed studies of dental anatomy and evolution in the initial chapter of synapsid evolution, when synapsids were able to evolve dentition for insectivory, herbivory, and carnivory. Numerous teeth, jaw elements, and skulls of the hypercarnivorous varanopid *Mesenosaurus efremovi* have been recently discovered in the cave systems near Richards Spur, Oklahoma, permitting the first detailed investigation of the dental anatomy of a Paleozoic tetrapod using multiple approaches, including morphometric and histological analyses. As a distant stem mammal, *Mesenosaurus* is the first member of this large and successful clade to exhibit a type of dental heterodonty that combines size and morphological (shape) variation of the tooth crowns. Here we present the first evidence of functional differentiation in the dentition of this early synapsid, with three distinct dental regions having diverse morphologies and functions. The quality and quantity of preserved materials has allowed us to identify the orientation and curvature of the carinae (cutting edges), and the variation and distribution of the ziphodonty (serrations) along the carinae. The shape‐related heterodonty seen in this taxon may have contributed to this taxon's ability to be a successful mid‐sized predator in the taxonomically diverse community of early Permian carnivores, but may have also extended the ecological resilience of this clade of mid‐sized predators across major faunal and environmental transitions.

## Graphical abstract

Here we present the first evidence of functional differentiation in the dentition of an early synapsid, *Mesenosaurus efremovi*. *Mesenosaurus* is the first member of this large and successful clade to exhibit a type of dental heterodonty that combines size and morphological (shape) variation of the tooth crowns, including variation and distribution of the ziphodonty (serrations) along the crowns cutting edges.

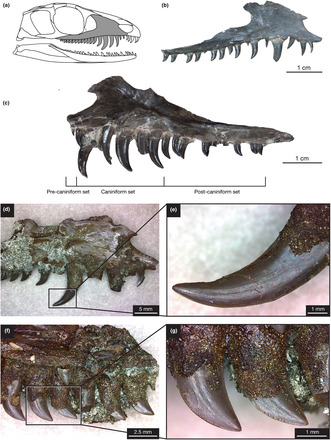

## 1 INTRODUCTION

Unravelling the evolution of the complex mammalian dentition from that of non‐mammalian early synapsids can offer insight into the feeding behaviours and ecologies of extinct taxa along the mammalian stem. One of the first major shifts in dental morphology among early synapsids toward mammals was an increase in dental differentiation, a condition known as heterodonty (Bels & Whishaw, 2019). Among predators, distinct and differently shaped teeth along the tooth row are likely to support different functions, such as capturing, disabling, defleshing, and even initial oral processing of prey (Ungar & Sues, 2019). While the mammalian pattern of heterodonty was first observed in early Mesozoic stem mammals (Kemp, 2005), less distinct forms of heterodonty have been observed in many early synapsids (Bels & Whishaw, 2019; Huttenlocker et al., 2021) and extant crocodiles (D'Amore et al., 2019). Dental heterodonty can be categorized as either size–shape heterodonty, which measures the variation in the different morphological characteristics of the tooth crown across each jawbone (Huttenlocker et al., 2021) or as functional heterodonty, which measures the amount of stress each particular tooth can transmit in relation to its specific position within the jawbone (Cohen, Weller, & Summers, 2020; Cohen, Weller, Westneat, et al., 2020). Recently, functional heterodonty has been found for various fish models (Cohen, Weller, & Summers, 2020; Cohen, Weller, Westneat, et al., 2020), and ongoing work on salamander dentitions (Huie & Cohen, 2023).

Ziphodont dentition, as seen in the extant apex predator *Varanus komodoensis*, is generally characterized by compressed, distally recurved, and serrated tooth crowns, but more specifically, true ziphodonty is a condition where the serrations along the carinae or cutting edges of the crown are composed of a dentine core underneath the enamel (Brink et al., 2015; D'Amore, 2009; D'Amore & Blumenschine, 2009). Ziphodont dentition has previously been shown to be efficient at processing prey and less prone to breakage (Abler, 1992; Brink et al., 2015; D'Amore, 2009; D'Amore & Blumenschine, 2009). In the fossil record, true ziphodonty is predominantly found in carnivorous dinosaurs (D'Amore, 2009; Torices et al., 2018; Whitney et al., 2020) throughout the Mesozoic and in the sphenacodontid *Dimetrodon* throughout the Permian (Brink & Reisz, 2014; Smith et al., 2005). The presence of heterodonty and ziphodonty plays a key role in interpreting an organism's feeding behaviour, as dental characteristics can be used to infer diet (Brink et al., 2015; Brink & Reisz, 2014; Friscia et al., 2007; Holliday & Steppan, 2004).

Among Pelycosaur‐grade synapsids, the highly carnivorous Varanopidae is the most widely dispersed, with a fossil record from the North American and European localities during the end of the Carboniferous and early Permian, and the Russian and South African localities during the late middle Permian (Maho et al., 2019; Reisz, 1986). Although there has been some suggestions that some or all varanopids may not be synapsids (Ford & Benson, 2020; Ivachnenko & Kurzanov, 1979; Reig, 1967), the most recent phylogenetic analysis places them back within Synapsida (Simões et al., 2022). The present study investigates the anatomy of the marginal dentition of the early Permian varanopid *Mesenosaurus efremovi* and describes it within the context of feeding strategies in this early terrestrial ecosystem. Within Varanopidae, *Mesenosaurus* is represented by an extensive collection of early Permian specimens from North America and late middle Permian specimens from Russia (Maho et al., 2019; Reisz & Berman, 2001). The temporal and geographical persistence of *Mesenosaurus* is unusual among Paleozoic amniotes and suggests a high degree of ecological resilience (Maho et al., 2019). *Mesenosaurus efremovi*, from the early Permian deposits of Richards Spur in Oklahoma, has previously been represented by three nearly complete skulls (Maho et al., 2019) as well as multiple isolated dental elements (Maho et al., 2022). While the cranial material allowed for a comprehensive study of the taxon, the initial description primarily focused on the visible external anatomy of the skulls. Therefore, the previously described cranial anatomy would be complemented by a comprehensive examination of the external and internal morphology of the dentition.

Such a study is made possible by the addition of new *Mesenosaurus efremovi* material, including three new skull fragments and 47 isolated dental elements. The present study describes, in detail, the variation in tooth size and gross morphology of the marginal dentition of *M. efremovi*. Additionally, through histological analysis, the dental and periodontal tissues of *M. efremovi* are identified, and true ziphodonty is confirmed within this early Permian amniote.

## 2 MATERIALS AND METHODS

The material used in the present study consists of three previously described skulls, three new skull fragments, and many new isolated premaxillae, maxillae, and dentaries, all assigned to *Mesenosaurus efremovi*. All of the specimens were collected from the early Permian Richards Spur locality (Dolese Brothers Limestone Quarry) near Fort Sill, Comanche County, Oklahoma. Previously described skulls (Maho et al., 2019) include OMNH 73209, the holotype (Figure S1), a laterally compressed, nearly complete skull and mandibles; OMNH 73208, a dorsoventrally compressed, nearly complete skull and mandible; and OMNH 73500, a laterally splayed, nearly complete skull and mandibles. Undescribed skull fragments include ROMVP 85440, a large block of fragmentary material with a laterally compressed, incomplete skull exposed in left lateral view and complete right dentary exposed in medial view; ROMVP 85441, an incomplete juvenile skull; and ROMVP 85439, the snout region of an incomplete skull. Isolated premaxillae, maxillae, and dentaries are listed in Table S1. Diane Scott conducted the preparation and photography of all specimens used in the study, and figures were prepared using Adobe Illustrator and Photoshop 2022.

Institutional abbreviations: OMNH, Sam Noble Oklahoma Museum of Natural History, University of Oklahoma, Norman, OK, USA; ROMVP, Royal Ontario Museum, Toronto, ON, Canada.

### 2.1 Morphological measurements

For all specimens listed above, the crown morphology at each tooth position was analyzed using crown size, crown elongation, crown basal shape, crown curvature, and denticle density (Figure S2), previously described by Smith et al. (2005). Digital images of the premaxillary, maxillary, and dentary marginal dentition were taken using Leica DVM6 digital microscope and LAS X software. Measurements from only fully erupted and complete crowns were attained through image analysis on ImageJ version 2.0.0. The size of crowns was assessed by measuring crown height (CH in mm), apical length (AL in mm), crown base length (CBL in mm), and crown base width (CBW in mm). Crown elongation was assessed by determining the crown height ratio (CHR = CH/CBL); larger crown height ratios corresponded with more elongated dentition. The labiolingual compression of the crown basal shape was assessed by determining the crown base ratio (CBR = CBW/CBL); crown base ratios closer to a value of 1 corresponded to round crown bases while smaller crown base ratios corresponded to more compressed or oval bases. Crown curvature was assessed by measuring the mid‐crown angle (CA in °) (Figure S3), which differs from Smith et al. (2005), which uses Law of Cosines and the crown basal angle; larger crown angles indicate that the crown apex is more centrally positioned and the mesial margins are less recurved, whereas lower crown angles indicate that the crown apex is positioned more posteriorly compared to the crown base and the mesial margins are more strongly recurved (Smith, 2005). To better visualize crown curvature patterns, the crown angle is plotted using the mean crown angles of 41 specimens (Figure S3a), and the individual crown angles of a single specimen (ROMVP 85440, Figure S3b) for each tooth position. Denticle density, the number of denticles along a carina per 0.5 mm, was determined for the mesial and distal carinae of the maxillary and dentary marginal dentition. To calculate denticle density, a distance of 0.5 mm was divided by the mean denticle size of each tooth margin, sampled near the apex, mid‐length, and base of the crown. The data cases for each variable measured were averages of one to seven replicate measurements on different specimens for each tooth position. Statistical analyses on every variable with respect to tooth position were run using R in RStudio version 2021.09.2 and illustrated using the ggplot2 package. A one‐way nested ANOVA test was also used to compare the means of every crown measurement with respect to the dental element (premaxilla, maxilla, and dentary), and accounted for having multiple crowns measured from the same specimen (Table 1). A two‐way ANOVA was also employed to compare the crown morphological measurements (CH, CBL, CBW, AL, CA) for the tooth positions within each jawbone separately (Table S2). The independent variable, tooth position, was squared since the data does not fit a linear model. Additionally, a backward selection model was implemented to remove the variables that had a *p*‐value of >0.1, such as Position^2^:Specimen and Position:Specimen for each of the crown measurments.

**TABLE 1:** Mean values and comparisons of the nine variables used in the study for the premaxillary (pm), maxillary (mx), and dentary (d) dentition of *Mesenosaurus efremovi* with standard deviation (SD). “pm‐mx” are *p* values of ANOVAs between premaxilla and maxilla values; “pm‐d” are *p* values of ANOVAs between premaxilla and dentary values; “mx‐d” are *p* values of ANOVAs between maxilla and dentary values.

Variable	Premaxilla (pm)	pm SD	Maxilla (mx)	mx SD	Dentary (d)	d SD	pm‐mx	pm‐d	mx‐d
Crown height (mm)	2.33	0.55	2.52	1.06	1.54	0.50	0.513	0.000353	<0.0001
Apical length (mm)	2.78	0.65	2.93	1.12	1.87	0.57	0.664	<0.001	<0.001
Crown base width (mm)	1.01	0.22	1.05	0.25	0.76	0.19	0.921	0.001	<0.001
Crown base length (mm)	1.14	0.28	1.67	0.41	1.10	0.22	<0.00001	0.968	<0.00001
Crown height ratio	2.06	0.24	1.52	0.44	1.41	0.42	<0.001	<0.001	0.0114
Crown base ratio	0.83	0.10	0.62	0.11	0.72	0.18	<0.001	0.112	<0.001
Crown angle (°)	142.67	3.61	140.72	7.72	142.06	7.39	0.472	0.572	0.973
Mesial denticle density	None	None	9.92	1.71	13.36	2.4	None	None	<0.00001
Distal denticle density	None	None	10.86	1.96	15.21	4.05	None	None	<0.00001

### 2.2 Neutron computed tomography

Neutron computed tomography (nCT) measurements of the holotype (OMNH 73209; Figure S1) were carried out at the Australian Nuclear Science and Technology Organisation (ANSTO). 16‐bit raw data was tomographically reconstructed, and virtual slices perpendicular to the axis of rotation were created. When these slices were stacked in sequence, they formed a three‐dimensional volume image of the specimen. The 16‐bit TIFF slices received from ANSTO were downsampled to 8‐bit in ImageJ, version 1.5.1, then rendered and segmented in Avizo Lite (Thermo Fisher Scientific; version 9.3.0).

### 2.3 Histology

Three types of thin sections of marginal dentition were constructed at the University of Toronto Mississauga: longitudinal anteroposterior (AP) section on ROMVP 85449; one longitudinal labiolingual (LL) section on ROMVP 85453; one transverse (TR) section on ROMVP 85453. Digital images of each specimen in labial and lingual views were taken using Leica DVM6 digital microscope and LAS X software. Measurements were obtained using image analysis on ImageJ version 2.0.0. The specimens were then individually embedded in Castolite AC polyester resin and placed inside a vacuum to harden. After the resin hardened, the specimens were cut using the Metcut‐5 low‐speed wafer blade saw (225 rpm), and the cut surfaces were polished using 1000‐grit silicon carbide paper with water as a lubricant. The polished surfaces of the specimens were then mounted on frosted plexiglass slides using super glue. Once the glue dried, the mounted specimens were cut again using the Metcut‐5, resulting in the mounted specimens having a thickness of approximately 1 mm. The mounted specimens were then ground to approximately 100 μm using a Metcut‐10 Geo thin sectioning machine grinding with a 30 μm grinding cup. Each thin section was further ground by hand using 1000‐, 1500‐, and 2000‐grit silicon carbide paper with water as a lubricant and hand polished using 1 μm grit aluminum oxide powder on a felt cloth. The thin sectioned specimens were photographed using a Nikon DS‐Fi1 camera mounted onto a Nikon AZ‐100 microscope with a magnification between 5 × 1 and 5 × 7. The photographs were examined using the NIS Elements‐Basic Research software. Histological characteristics were examined with a focus on dental and periodontal tissue anatomy.

## 3 RESULTS

### 3.1 External dental anatomy

**FIGURE 1:**
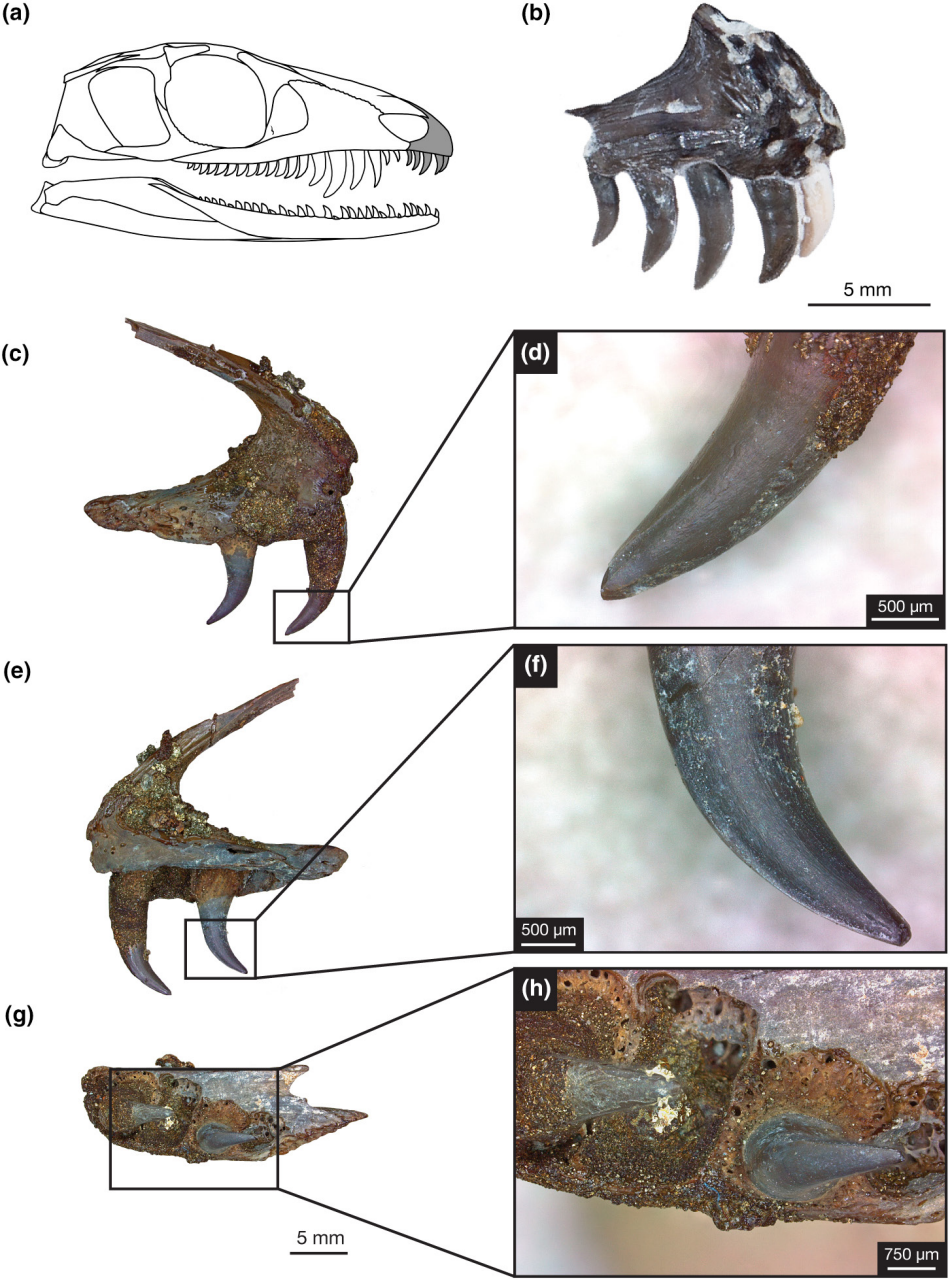
Premaxillary dentition of *Mesenosaurus efremovi*. (a) Skull drawing of *M. efremovi* highlighting premaxilla. (b) Photograph of OMNH 73500 premaxilla in right lateral view. Photographs of ROMVP 85458 in (c) labial view. (d) Closeup of labial view showing distal carinae. (e) Lingual view. (f) Closeup of lingual view showing mesial carinae. (g) Ventral (occlusal) view. (h) Closeup ventral view showing alveoli. Skull drawing was outlined and modified from Maho et al. (2022).

**FIGURE 2:**
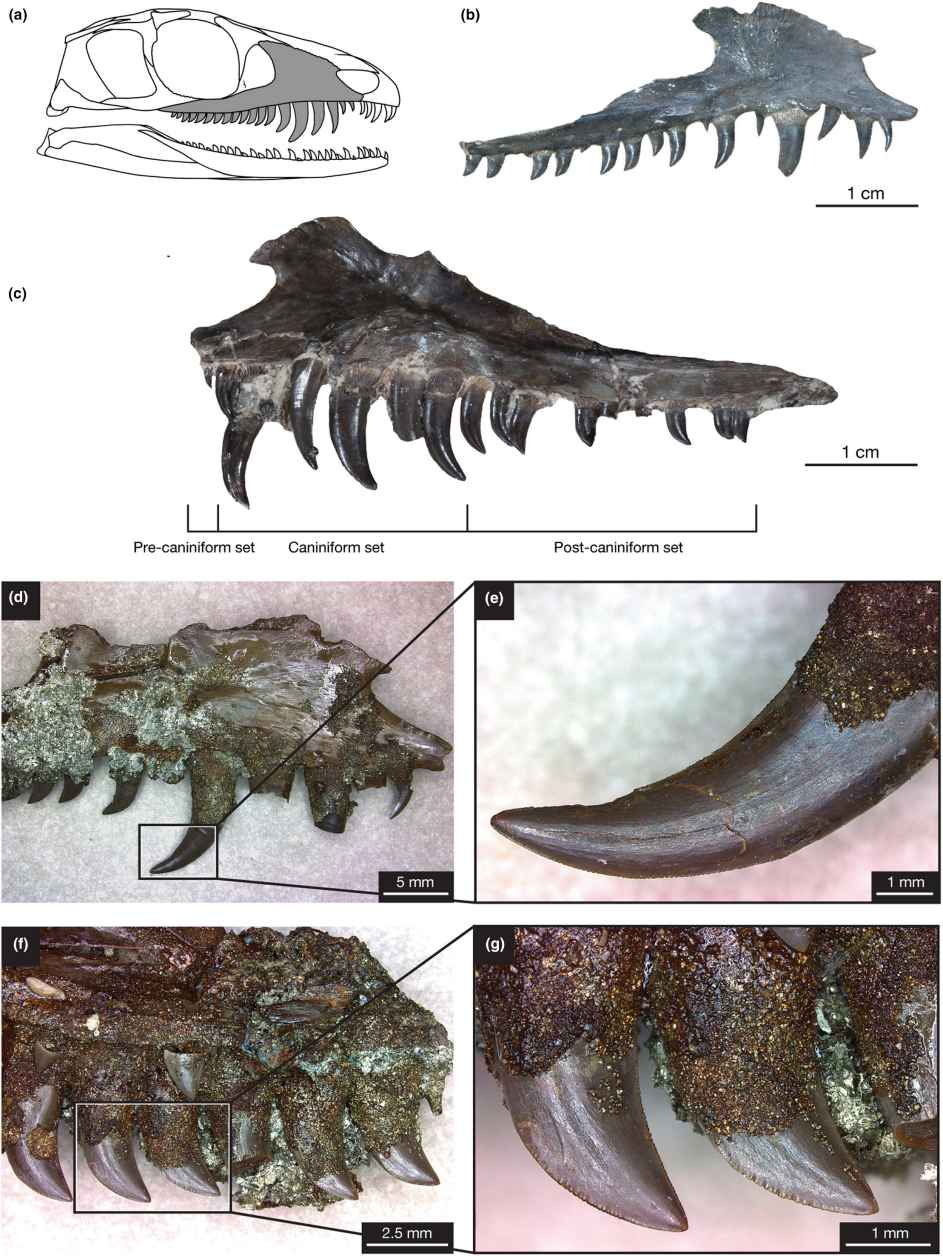
Maxillary dentition of *Mesenosaurus efremovi*. (a) Skull drawing of *M. efremovi* highlighting maxilla. (b) Photograph of OMNH 73208 maxilla in right lateral view. (c) Photograph of ROMVP 85440 maxilla in left lateral view showing pre‐caniniform, caniniform, and post‐caniniform regions. (d) Photograph of ROMVP 85457 in labial view. (e) Closeup view showing serrated mesial and distal carinae of caniniform dentition. (f) Lingual view. (g) Closeup view showing serrated mesial and distal carinae of post‐caniniform dentition. Skull drawing was outlined and modified from Maho et al. (2022).

**FIGURE 3:**
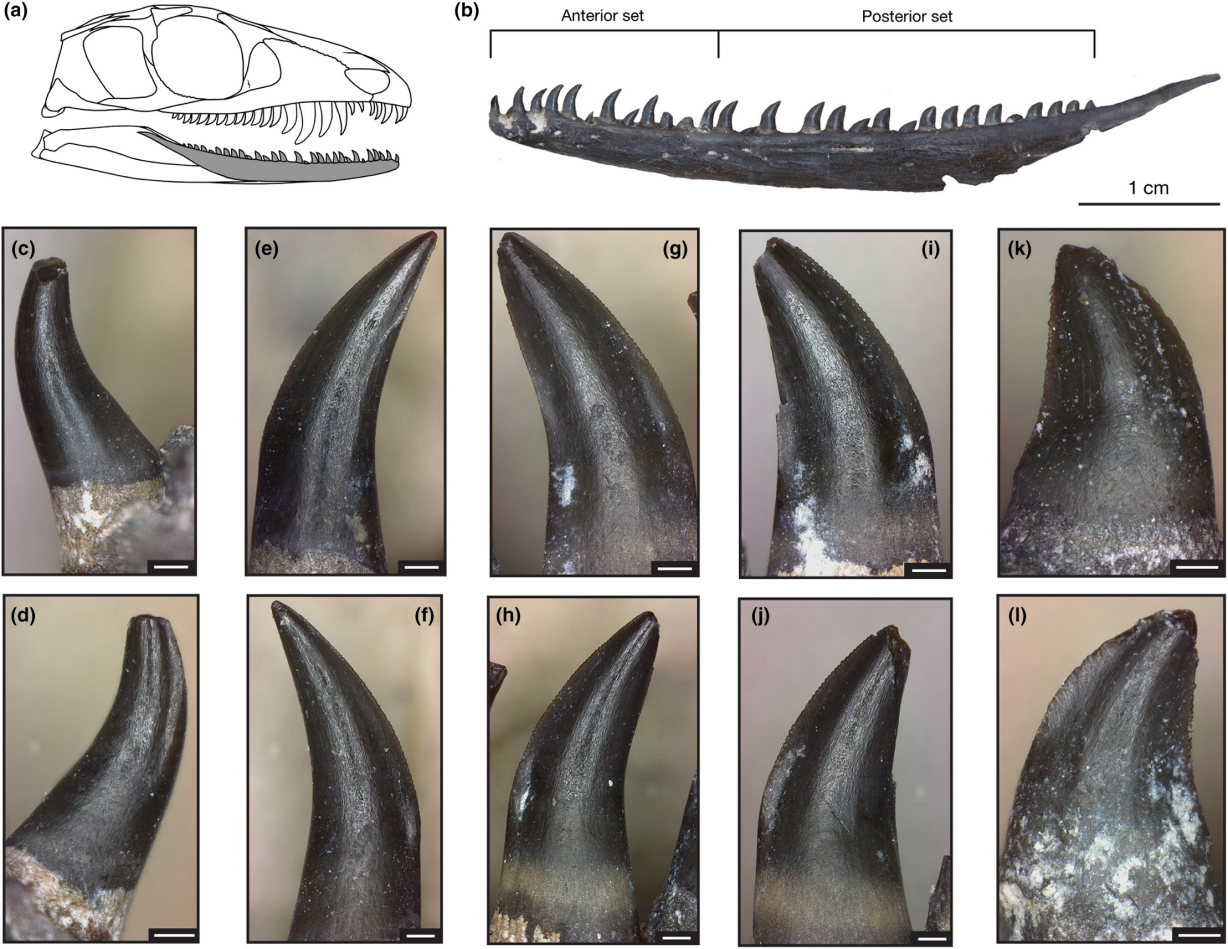
Dentary dentition of *Mesenosaurus efremovi*. (a) Skull drawing of *M. efremovi* highlighting dentary. (b) Photograph of OMNH 73208 left dentary in lateral (labial) view showing mesial and distal regions. ROMVP 85492, tooth position 1 in (c) labial view and (d) lingual view. Tooth position 3 in (e) labial view and (f) lingual view. ROMVP 85499, tooth position 13 in (g) labial view and (h) lingual view. Tooth position 17 in (i) labial view and (j) lingual view. ROMVP 85498, tooth position 26 in (k) labial view and (l) lingual view. Scale = 250 μm. Skull drawing was outlined and modified from Maho et al. (2022).

The marginal dentition of *Mesenosaurus efremovi* (Figures 1, 2, 3) can generally be described as being strongly recurved and labiolingually compressed, with a maxillary caniniform region with a series of enlarged teeth, serrated cutting edges, and no occlusal surfaces. However, further variation is observed between and within the premaxillary, maxillary, and dentary tooth series. *M. efremovi* possesses diagnostic dental characteristics of varanopids, including the presence of round tooth bases in cross‐section, labiolingual compression towards the crown apex, and strong crown recurvature (Berman & Reisz, 1982; Langston & Reisz, 1981). The presence of serrations/denticles on the cutting edges of the marginal teeth is not a diagnostic characteristic in varanopids; however, *M. efremovi* shares this characteristic with *Heleosaurus*, *Mycterosaurus*, and *Elliotsmithia* (Reisz et al., 1998; Reisz & Berman, 2001; Reisz & Modesto, 2007). However, serrated cutting edges are absent in *Varanops*, *Varanodon*, and *Aerosaurus* (Modesto et al., 2011).

### 3.2 Premaxilla

The premaxillary tooth series (Figure 1) of *Mesenosaurus efremovi* contains five tooth positions in all known premaxillae (Table S1). Having five tooth positions is consistent within the genus *Mesenosaurus*, which includes *M. romeri* (Reisz & Berman, 2001) while varying between three to six tooth positions amongst other varanopids (Brocklehurst et al., 2016; Campione & Reisz, 2010; Langston & Reisz, 1981; Reisz & Dilkes, 2003; Spindler et al., 2018). In palatal view, the tooth row of *M. efremovi* curves distally away from the mid‐sagittal line of the premaxilla, along the ventral margin (Figure 1) due to the anterior end of the snout being squared off. The alveoli (tooth sockets) of the premaxilla are closely spaced, oval in shape, and obliquely angled posteromedially. Labial surface of the premaxillary crowns are adjacent to the ventral margin of the premaxilla.

**FIGURE 4:**
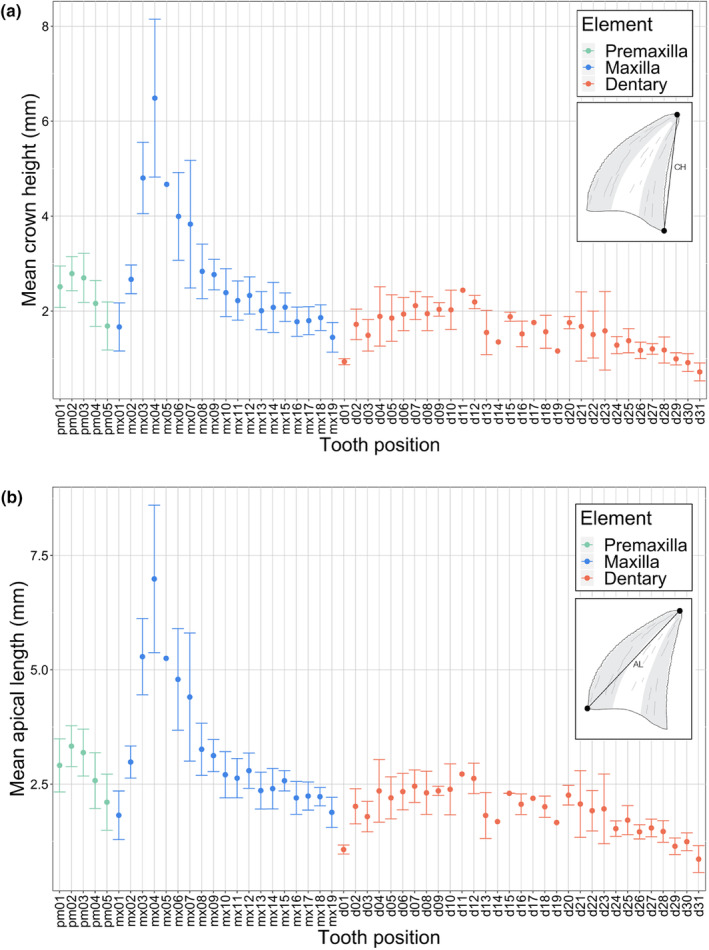
Crown size (in mm) variability profiles with respect to tooth position for premaxillary, maxillary, and dentary dentition of *Mesenosaurus efremovi*. (a) Mean crown height (CH), *n* = 41. (b) Mean apical length (AL), *n* = 41.

**FIGURE 5:**
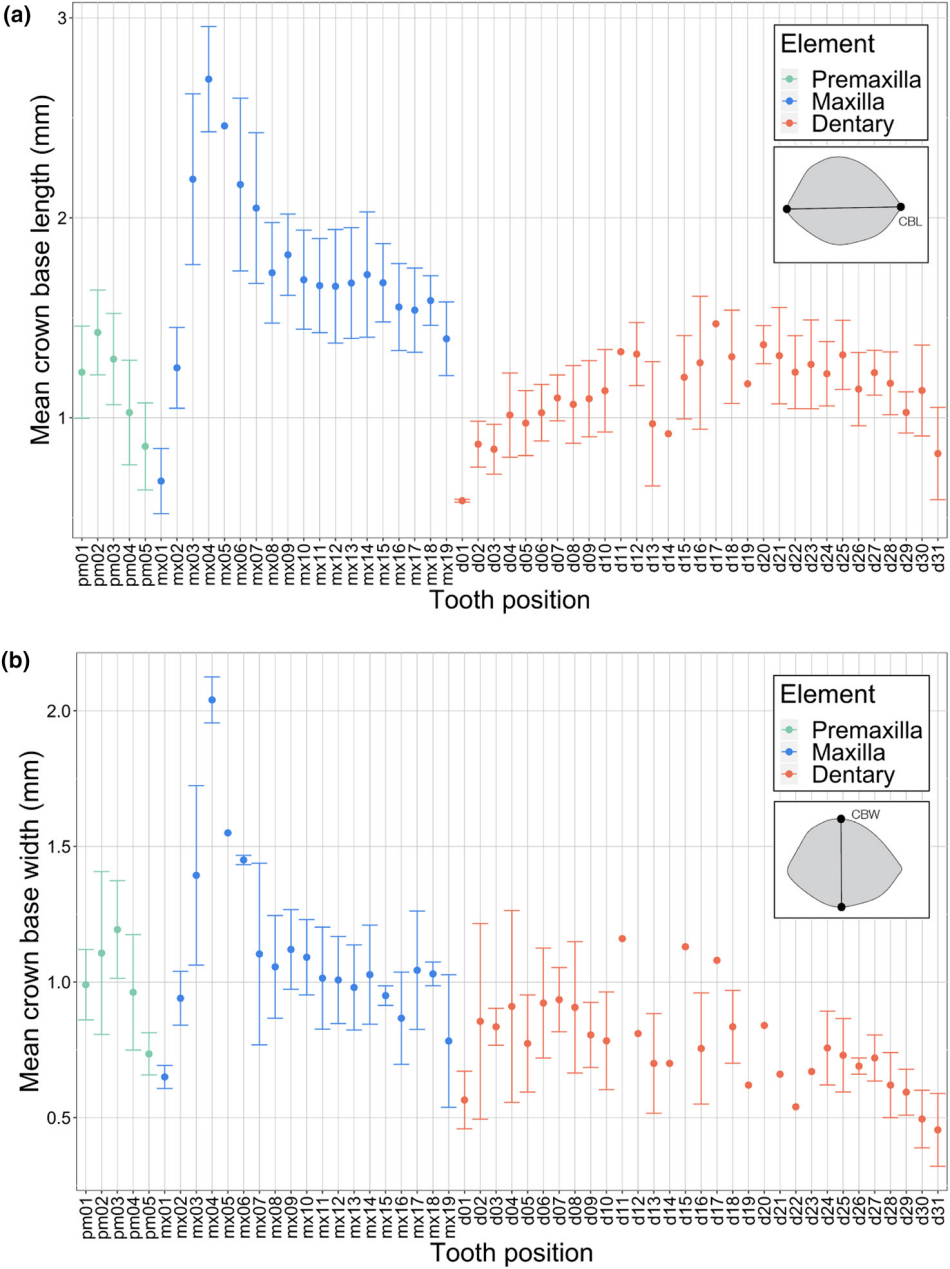
Crown base size (in mm) variability profiles with respect to tooth position for premaxillary, maxillary, and dentary dentition of *Mesenosaurus efremovi*. (a) Mean crown base length (CBL), *n* = 41. (b) Mean crown base width (CBW), *n* = 41. Green represents the premaxilla, blue represents the maxilla, and red represents the dentary.

Similar patterns for variation in crown size are observed amongst the four variables (CH, AL, CBL, and CBW) examined at each tooth position (Figures 4 and 5); therefore, mean crown height is utilized in the following description as the measure for tooth size. Tooth crowns at the first position (pm01), second position (pm02), and third position (pm03) are similar in height, 2.51 ± 0.44, 2.70 ± 0.52, and 2.79 ± 0.36 mm, respectively. The crowns at pm02 and pm03 are typically the largest of the premaxillary series (Figures 1b and 4a). Tooth crowns at the fourth position (pm04) and fifth position (pm05) are smaller, decreasing in size distally, 2.36 ± 0.39 and 1.94 ± 0.37 mm, respectively (Figures 1a and 4a). The premaxillary crown bases are round in shape with tapered tips in the mesial and distal margins, corresponding to the mesial and distal carinae locations. The crown base's long axis, measured as the crown base length (Figure 1b,c), is oriented mesiodistally along the ventral margin of the premaxilla, curving away from the mid‐sagittal line distally. Similar to crown size, the crowns at pm03 are typically the most elongated of the premaxillary tooth row. The labiolingual compression, or roundness of the crown bases, is analyzed by determining the crown base ratio, with the crowns at pm03 typically being the roundest, 0.93 ± 0.05, while the crowns become more compressed distally, corresponding to smaller values of the mean crown base ratio (Figure 3b). Crowns at pm01 and pm02 have smaller crown angles than crowns at pm03, pm04, and pm05, demonstrating a decrease in crown curvature and apex displacement distally (Figure S3a).

The ANOVA revealed no significant differences for each of the crown morphological variables (Table S2).

### 3.3 Maxilla

The maxillary tooth series (Figure 2) of *Mesenosaurus efremovi* at an adult stage typically contains 19 tooth positions; however, a juvenile specimen (ROMVP 85441) has 21 tooth positions (Table S3). This trend of decreased tooth positions with ontogeny can be unusual among extinct tetrapods (Brown et al., 2015), however, some taxa have been shown to exhibit this trend (Davit‐Béal et al., 2009; Haridy et al., 2018). The maxillary tooth series exhibits greater variation in tooth size and gross morphology than the premaxillary series and, is divided into three distinct sets: a pre‐caniniform set (mx01 to mx02), a caniniform set (mx03 to mx06), and a post‐caniniform set (mx07 to mx19; Figure 2c). The alveoli of the maxilla are closely spaced along the ventral margin and round in shape (Figure S1d). The labial crown surfaces are adjacent to the ventral margin of the maxilla, whereas the lingual surfaces are located between the ventral and medial margins, providing space for successive replacement teeth to develop lingually (Figure S1d). The maxillary crowns at mx01 and mx02 are comparable in size to the premaxillary crown, with a mean crown height, apical length, crown base width, and crown base length falling within the range of the premaxillary dentition (Figures 4 and 5). There is an increase in tooth size (crown height) distally from mx01 to mx04, 1.67 ± 0.51 to 6.28 ± 2.30 mm, with crowns at mx04 typically being the largest of the maxillary series (Figure 4a). Similarly, this trend is observed for the crown base width and length, with mx04 having the largest CBW (Figure 5a) and CBL (Figure 5b). There is an inflection point at mx04, where crown height decreases distally towards the end of the tooth row, with mx19 having the smallest crown height, 1.44 ± 0.31 mm (Figure 4a).

**FIGURE 6:**
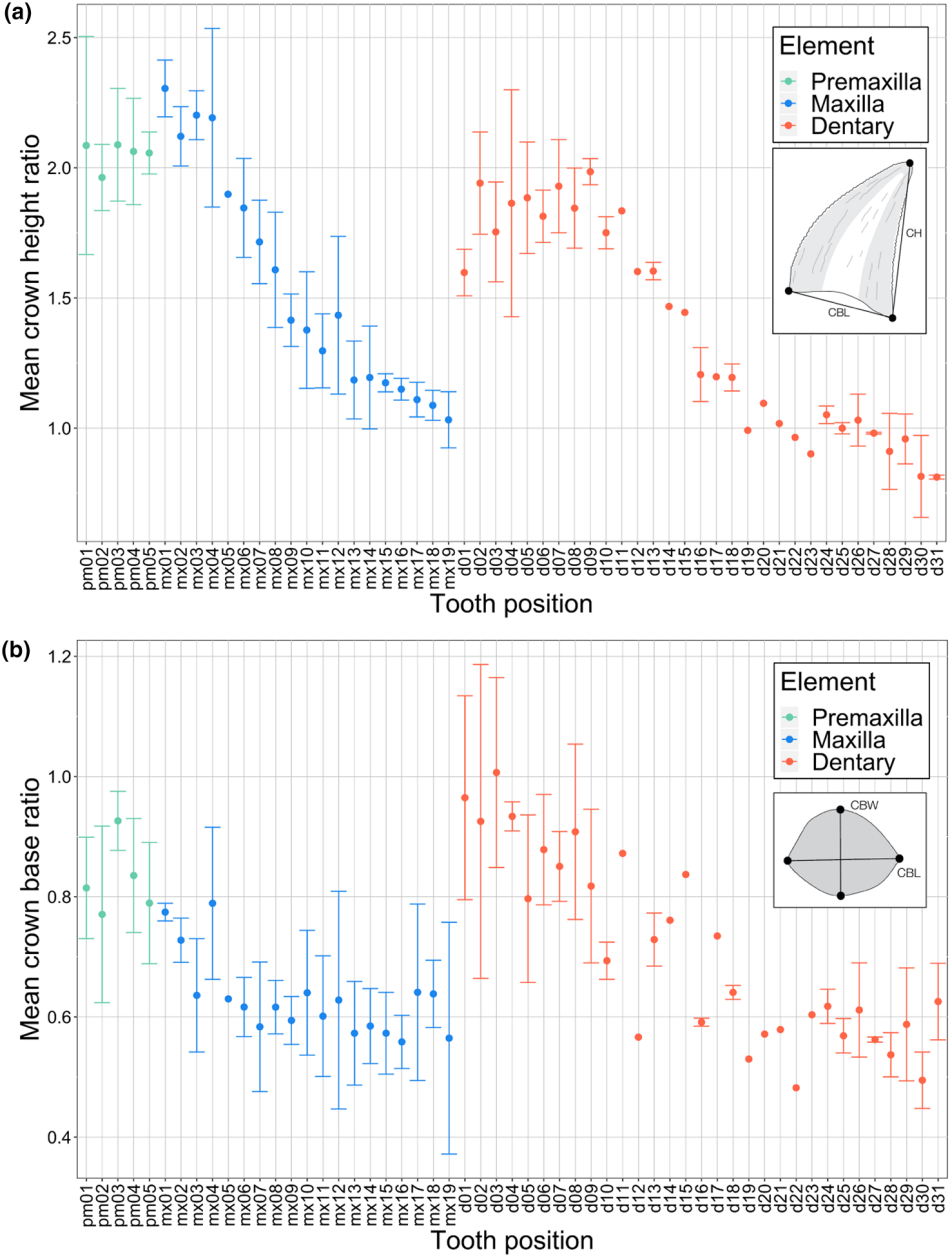
Crown shape variability profiles with respect to tooth position for premaxillary, maxillary, and dentary dentition of *Mesenosaurus efremovi*. (a) mean crown height ratio (CHR), *n* = 41. (b) mean crown base ratio (CBR), *n* = 41. Green represents the premaxilla, blue represents the maxilla, and red represents the dentary.

The caniniform region varies with ontogeny; smaller (presumably less mature specimens) have four caniniform teeth, mx03 to mx06 (OMNH 73208, Figure 2b), while more mature specimens have five caniniform teeth, mx03 to mx07 (ROMVP 85440, Figure 6c). Throughout ontogeny, the crown at mx07 appears to increase in size and become a caniniform tooth, pushing the pre‐caniniform teeth mesially while the number of tooth positions and the size of the other teeth remaining constant. The long axis of the crown base (CBL) is oriented mesiodistally, parallel with the ventral margin of the maxilla. Crown elongation appears to be reduced distally, with the maxillary crowns being significantly less elongated than the premaxillary crowns (Table 1; Figure 6a). The maxillary crown at mx01 is the most elongated in shape, having the largest mean CHR of the upper tooth row, 2.30 ± 0.11 (Figure 6a). The maxillary crowns become increasingly less elongated distally, with mx19 having the smallest CHR, 1.03 ± 0.11, (Figure 6a). Compared to the premaxillary dentition, the maxillary crown bases are significantly more labiolingually compressed (Table 1; Figure 6b). The mean CBR of the maxillary tooth series ranges from the roundest base at mx04 to the most compressed base at mx19 (Figure 2b). Generally, the pre‐caniniform and caniniform maxillary crown bases are rounder than the post‐caniniform crown bases, demonstrating a basal shape trend of increased labiolingual compression distally in the maxillary tooth row (Figure 6b).

The crown angles appear to decrease distally in the maxillary tooth series (Figure S3), indicating more displaced apices and an increase in curvature of the mesial margins distally. The pre‐caniniform and caniniform maxillary crowns have mesial and distal margins that rise apically with a smooth curve from the base to the apex (Figure 2b–e). The post‐caniniform crowns have mesial margins that, instead, exhibit a sharp curve below the mid‐crown height towards the apex, corresponding to the length of the mesial carinae and resulting in the mesial cutting edges becoming the ventral edges of the crown. The distal margins of the post‐caniniform crowns become increasingly more straightened distally (Figure 2b,c,f,g).

The ANOVA revealed significant differences in only three of the crown morphological variables, CH, CBL, and CA, when comparing each of the tooth positions within the various maxilla specimens (Table S2).

### 3.4 Dentary

The dentary tooth series (Figure 3) of *Mesenosaurus efremovi* contains between 30 and 31 teeth (Table S1), typical amongst varanopids (Campione & Reisz, 2010; Reisz & Berman, 2001; Reisz & Dilkes, 2003). While the dentition of the lower jaw does not exhibit as much variation as the upper marginal dentition in terms of tooth size and gross morphology, the dentary teeth can be divided into two sets: the anterior set (d01 to d11), and the posterior set (d12 to d31), based on inflection points in the morphological trends observed (Figures 3b, 4 and 6a). The anterior set resemble the dentition of the premaxilla and pre‐caniniform dentition of the maxilla, while the posterior set resembles the post‐caniniform dentition of the maxilla. Similar to the maxilla, the alveoli are closely spaced and round in shape (Figure S1e). The labial surfaces are adjacent to the ventral margin of the dentary, whereas the lingual surfaces are between the ventral and medial margins, providing space for successive replacement teeth to develop lingually (Figure S2e). Overall, the dentary contains the smallest crowns of the marginal dentition with a mean crown height, apical length, crown base width, and crown base length falling below the range of the premaxillary and maxillary dentition (Table 1; Figures 4 and 5). There appears to be an increase in tooth size distally from d01 to d11, 0.94 mm to 2.44 mm (Figure 4a). In terms of crown height (CBH and CAH), d11 is the largest tooth in the dentary series, which is also considered to be the last tooth in the anterior set because it marks the inflection point. Beyond this point, the crown height decreases distally towards the end of the tooth row, with teeth at d31 having the smallest crown heights, 0.72 ± 0.19 mm (Figure 4a). Like the maxilla, the long axis of the crown base (CBL) is oriented mesiodistally, parallel to the dentary's ventral margin. Overall, the dentary crowns are significantly less elongated than the premaxillary crowns and more similar in elongation to the more distal maxillary crowns (Table 1). Two trends were observed in terms of crown elongation; first, the anterior set appears to increase in elongation distally, ranging from 1.60 ± 0.09 to 1.98 ± 0.05, and second, the posterior set appears to decrease in elongation distally, ranging from 1.60 ± 0.09 to 0.81 ± 0.01 (Figure 6a).

Overall, the dentary crowns are more labiolingually compressed than the premaxillary crowns and less compressed than the maxillary crowns (Figure 3). Like the upper marginal dentition, the lower marginal dentition exhibits a basal shape trend of increased compression distally (Figure 2b). The mean CBR ranges from the roundest bases at d03 to the most compressed bases at d30 (Figure 2b). A clear trend is not observed for the mean crown angle in the dentary tooth series, possibly due to too much variation (Figure S3a). However, when observing a single specimen (ROMVP 85440; Figure S3b), a trend of decreasing crown angle distally is observed, indicating more recurved mesial margins distally. Within the anterior set, the crowns have mesial and distal margins that rise apically with a smooth curve from the base to the apex (Figure 3c–l), whereas the crowns in the posterior set, have mesial margins that, like in the maxilla, exhibit a sharp curve between the base and the mid‐crown height towards the apex, corresponding to the length of the mesial carinae and resulting in the mesial cutting edges becoming the dorsal edges of the crown. The distal margins become increasingly straightened distally.

The ANOVA revealed significant differences in all of the crown morphological variables when comparing each of the tooth positions within the various dentary specimens (Table S2). However, significant differences were also observed when comparing specimens to one another and when examining if an interaction exists between the two independent variables, tooth position and specimen. Therefore, the degree of difference for each of the individual crown morphology measurements varies across individual specimens per tooth position.

### 3.5 Morphology of carinae and ziphodonty

The premaxillary dentition does not exhibit ziphodonty on the mesial or distal margins of the crown. Rather than serrations, a thin carina (cutting edge), appearing as a narrow ridge, is present on both the mesial and distal margins, which begins at the crown apex and extends basally. The mesial carina appears to extend farther down toward the base of the tooth crown (Figure 1f), compared to the distal carina, which terminates either close to or slightly above the mid‐crown (Figure 1d). Neither carinae extend throughout the entire edge of the tooth. The premaxillary carinae are situated at the linguomesial and labiodistal corners of the crowns; mesial carinae are visible on the lingual side (Figure 1e,f), while distal carinae are visible on the labial side (Figure 1c,d). This morphology is seen in all five premaxillary teeth, including new replacement teeth that are growing and in the process of attachment.

Similar to the premaxillary dentition, the first maxillary tooth (mx01) has a carina on the mesial margin, visible on the lingual side, with no serrations (denticles) present on the cutting edge, whereas the distal margin of mx01 exhibits a very short carina, lacking serrations. The third maxillary tooth (mx03), larger in size than the first, exhibits serrations on both the mesial and distal carinae. The maxillary carinae are situated more mesiodistally than the premaxillary carinae, parallel to the long axis of the crown base. The mesial carinae are shorter than the distal carinae, terminating between the mid‐crown height and crown base, where the sharp curve occurs, while the distal carinae terminate at the crown base. Additionally, cutting edges expand slightly outward from both tooth margins within the basal portion of the serration carinae, creating a larger surface area. An increase in the amount and size of the denticles is observed within the caniniform region (mx04 to mx06) (Figure 2e), with a slight decrease in the amount of denticles distally in the post‐caniniform region. The posterior maxillary teeth, sequentially decreasing in tooth height, appear to have serrations on both the mesial and distal margins, with a greater amount on the distal margin extending throughout the entire crown (Figure 2g). Throughout the crowns of the caniniform and post‐caniniform regions, the largest denticles on both the mesial and distal carinae are observed at the mid‐crown height, decreasing in size towards the crown apex and base.

The lower jaw dentition exhibits only carinae with no serrations present on the most anterior teeth. The carinae for the first dentary tooth (d01) appear as a thin, narrow ridges that are positioned linguomesially and labiodistally, similar to the premaxillary dentition (Figure 3c,d). The third tooth (d03) has a short carina on the distal margin, extending from the crown apex labially towards the mid‐crown where it terminates, whereas the mesial margin of d03 appears to have slight serrations present on the cutting edge that are not well‐defined with no interdental sulci present between denticles, and overall small in size (Figure 3e,f). By the fifth tooth (d05), the small carina on the distal cutting edge exhibits small serrations on the top half, while the bottom half appears as a thin ridge which extends further labially on the mid‐crown. Further posteriorly in the tooth row (d07 and d09), serrations present on the mesial margin have increased in size and appear better developed with interdental sulci present, and an outward expansion of the cutting edge is observed at the crown base, whereas the distal cutting edge is extending further basally as the tooth height increases posteriorly. The dentition located in the middle region of the dentary exhibits a similar ziphodont morphology (Figure 3i–l).

Like the premaxillary crowns, the dentary crowns of the anterior set have distal carinae that are shorter than the mesial carinae, terminating near the mid‐crown height compared to the mesial carinae, which terminate closer to the base. This is unlike the maxillary dentition that exhibits serrations extending throughout the entire distal edge of the crown. The crowns of the posterior set are more like the maxillary crowns, exhibiting mesial carinae that are shorter than the distal carinae, terminating between the mid‐crown height and crown base, while the distal carinae terminate closer to the crown base (Figure 3k,l). Qualitatively, the distal crowns exhibit more distinct denticles, and within each crown, the largest denticles on both carinae are observed at the mid‐crown height.

Overall, the shape, frequency, and location of ziphodonty vary along the tooth row of *M. efremovi*. The serrations appear absent or modest along the anterior region, becoming well‐developed on the teeth that are large with great recuvature. In the posterior region of the marginal dentition, where the teeth are recurved, there is an increase in the density of serrations near the tooth apex and throughout the length of both tooth edges.

### 3.6 Dental microanatomy

**FIGURE 7:**
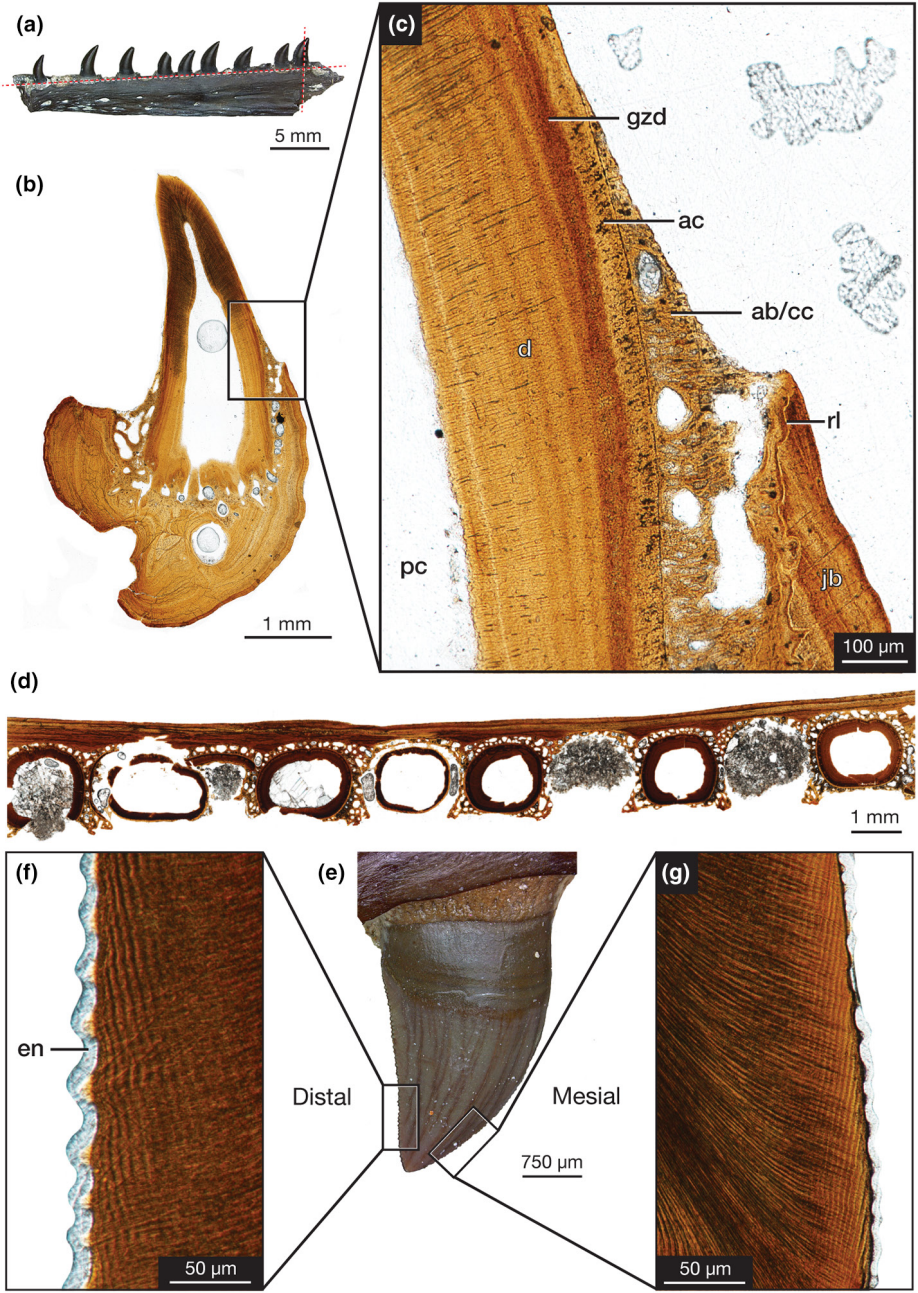
Microanatomy of *Mesenosaurus efremovi*. ROMVP 85453, dentary tooth showing (a) Photograph in labial view showing planes of TR cross‐section and longitudinal LL sections. (b) Whole view of tooth longitudinal LL section. (c) Closeup view of attachment tissues in longitudinal LL section. (d) Whole view of dentary TR cross‐section. ROMVP 85449, maxillary ziphodont teeth showing (e) photograph in labial view. Longitudinal AP section of (f) distal carina and (g) mesial carina. ab alveolar bone; ac, acellular cementum; cc, cellular cementum; d, dentine; en, enamel; gzd, globular zone of dentine; jb, jawbone; pc, pulp cavity; rl; reversal line. Figure concept after LeBlanc et al. (2016).

A partial dentary was sectioned for analysis of the dental microanatomy of *Mesenosaurus efremovi* (Figure 7). Tooth implantation in *M. efremovi* appears to be subthecodondont, whereby the teeth are implanted into sockets and ankylosed to the jaw. Dental ankylosis is a condition where the dental mineralized tissue of the tooth is fused to an attachment bone, which is fused to the jawbone (Figure 7b) (Bertin et al., 2018). This condition is prevalent throughout the fossil record of early reptiles and early synapsids (Edmund, 1960; Gaengler, 2000; LeBlanc & Reisz, 2013).

Histological sections of the tooth root (Figure 7c,d) reveal dental and periodontal tissues consistent with other early synapsids and similar to a previously documented unspecified varanopid from the Richards Spur locality (LeBlanc et al., 2018). The dentine is coated by a thin band of light‐colored tissue (Figure 7c), acellular cementum. The acellular cementum separates the dentine root from a microcancellous bone tissue, comprising of cellular cementum and alveolar bone, indistinguishable from each other due to the lack of periodontal space. This tissue has a fibrous texture that is oriented perpendicular to the tooth's vertical axis and consists of osteocyte lacunae that are larger and more disorganized than those within the jawbone (Figure 7c). A reversal line separates this tissue from the jawbone and indicates that cellular cementum and alveolar bone are continuously resorbed and redeposited with each replacement event (LeBlanc et al., 2018). Interestingly, the acellular cementum and the microcancellous bone extend apically beyond the jawbone towards the base of the tooth crown (Figure 7c). The tooth crown tissues consist of dentine, with visible dentinal tubules extending from the pulp cavity to the dentine's exterior and oriented perpendicular to the dentine's deposition plane parallel to the enamel‐dentine junction, and a thin layer of enamel covering the crown (Figure 7).

A histological longitudinal AP section of the denticles on the mesial and distal cutting edges of the crown shows the presence of a form of true ziphodonty, serrations composed of a layer of enamel over a dentine core (Figure 7e–g). Both the mesial and distal cutting edges exhibit unevenly sized denticles, smaller near the base and apex of the crown, and larger near the mid‐crown height, covered by an even layer of enamel. Interdental folds (ampullae) are not present between adjacent denticles.

## 4 DISCUSSION

The variation in the dentitions of different terrestrial amniotes is strongly correlated with variation in their ecologies. Due to this tight correlation, dental characteristics may be used to infer aspects of an organism's diet or ecology (Brink & Reisz, 2014; Friscia et al., 2007; Holliday & Steppan, 2004). For example, dentition with a relative increase in grinding surfaces reflects a highly herbivorous or omnivorous diet, whereas dentition with a relative increase in shearing surfaces reflects a highly carnivorous diet (Van Valkenburgh, 1989). The dentition of *Mesenosaurus efremovi* appears to have similar dental characteristics of other varanopids (Berman & Reisz, 1982; Langston & Reisz, 1981). Exceptions to these characteristics are observed in *Pyozia* and *Archaeovenator*, varanopid taxa with teeth that lack labiolingual compression and a maxillary caniniform region; they may represent the primitive conditions for the family (Anderson & Reisz, 2004; Reisz & Dilkes, 2003). The strongly recurved, labiolingually compressed, and serrated crowns of *Mesenosaurus efremovi* reflect a highly carnivorous diet, likely even hypercarnivorous (a diet consisting of mostly meat; Maho et al., 2022), with a diet comprising most likely of other tetrapods. While most varanid lizards, which share a considerable resemblance to varanopids, prey on a variety of invertebrates and vertebrates, adult *Varanus komodoensis*, *V. glebopalma*, and *V. griseus* exhibit almost exclusive meat‐only diets, preying mainly on vertebrates (Holliday & Steppan, 2004; Losos & Greene, 1988; Pianka et al., 2004; Williston, 1914).

Varanid lizards represent an extant taxon that parallels the morphology of varanopids (Maho et al., 2022); thus, recent observations on the hypercarnivorous feeding behaviours of varanid lizards (D'Amore & Blumenschine, 2009) may be used to infer such behaviours in *M. efremovi*. The upper and lower teeth of *Mesenosaurus* are laterally compressed and blade‐like, and are similar to the hypercarniverous *V. komodoensis*. *Varanus exanthematicus* exhibits a distinctly different pattern, where only the anterior‐most upper and lower teeth are slender and slightly recurved, and the posterior teeth are laterally widened and peg‐like (T.M. personal observations). The crown length and height of *M. efremovi* teeth show a distinct trend anteroposteriorly, where the anterior teeth are significantly longer and thinner than the short and wide posterior teeth.

Dental heterodonty is a combination of morphology (shape) and size, including the crown height and the length and width of the crown base. Size heterodonty is most apparent within the maxillary dentition, with the pre‐caniniform teeth being small, followed by a dramatic increase in the size in the caniniform region, with a gradual decrease posteriorly in size for the post‐caniniform region (Figure 4a). In addition to the obvious variation in size, shape heterodonty is also a prominent feature in *Mesenosaurus efremovi* through variation in the shape of the dentition, the orientation and disposition of carinae on the crown cutting edges, and the distribution of serrations along the carinae. Variation in ziphodonty, including its presence or absence, and the morphology of the individual denticles, indicates that each portion of the jawbone may have had a different function while feeding. Thus, the morphological and size variation of the teeth of *M. efremovi* provides strong support for the division of function along the upper and lower tooth rows, and suggests that there may have been extensive food manipulation in the mouth.

The Nile monitor, *Varanus niloticus*, secures prey items with needle‐like mesial teeth that do not greatly change shape from juvenile to adult stages, likely reflecting the conservation of function throughout ontogeny. Through intraoral transportation, juvenile *V. niloticus* moves prey items towards more blade‐like distal teeth that function in processing. Similarly, the elongated dentition and absence of serrations within the carinae of the premaxilla, the pre‐caniniform region of the maxilla, and the anterior‐most dentition within the mesial region of the dentary in *M. efremovi* suggest that these anterior regions may have functioned in grabbing and piercing prey. While the caniniform region, which has the largest and most elongated crowns of the marginal dentition, likely functioned in prey disablement, these teeth may have also been engaged in defleshing behaviour, as evidenced by the high concentration of serrations on the distal edge. This function is reflected in the strength of these crowns: reinforced by the microcancellous bone extending past the jawbone, deep tooth implantation, and the massive reinforcement of the maxillary bone in this region. The labiolingually compressed dentition of the post‐caniniform region of the maxilla and distal region of the dentary in *M. efremovi* likely functioned in cutting and further defleshing prey, as the mesial margins were recurved to create ventral and dorsal edges of the crowns, respectively, in addition to the high concentration of serrations observed on both crown edges, for greater contact with prey items. While some similarities between the division of function along the marginal dentition in *V. nicoticus* and *M. efremovi* are apparent, it must be noted that adult specimens of *V. niloticus* further develop bulbous distal teeth that can also function as grinding or crushing surfaces (D'Amore, 2015; Estes & Williams, 1984). This morphology is not observed in *M. efremovi*, which has no occlusal and grinding surfaces.

Similar to varanid lizards, the presence of ziphodonty in *M. efremovi* may have facilitated more efficient feeding by reducing the energy required to penetrate and slice through prey (D'Amore & Blumenschine, 2009). Additionally, *M. efremovi* was found to have an exceptionally rapid tooth replacement rate which was suggested to facilitate the maintenance of a proper set of teeth for feeding (Maho et al., 2022). This study presents the first occurrence of true ziphodonty in this clade; in the fossil record, true ziphodonty is predominantly found in carnivorous archosaurs throughout the Mesozoic (Brink et al., 2015; Whitney et al., 2020), and in the apex predator *Dimetrodon* during the early Permian (Brink & Reisz, 2014; Smith et al., 2005). However, the morphology of the serrations present in some species of *Dimetrodon* differs from those in *M. efremovi*, and the absence of serrations in other varanopid taxa suggests that serrations in these two early Permian synapsids are not homologous. Ziphodont crowns in the extant *V. komodoensis* are more effective in defleshing and cutting prey items than non‐ziphodont crowns of other varanids (D'Amore & Blumenschine, 2009). Similarly, the recurvature and serrations of ziphodont crowns in *M. efremovi* suggest that these crowns were effective in biting and pulling but were not likely used to crack bone. The presence of ziphodonty may have enabled *M. efremovi* to feed on larger prey items and may have been important in facilitating a hypercarnivorous feeding style (Abler, 1992; Brink & Reisz, 2014; De Andrade et al., 2010).

*Mesenosaurus* is a particularly interesting Paleozoic amniote because it has a long evolutionary history, with a fossil record extending from the early Permian of North America into the late middle Permian of Russia, and extending beyond Olson's gap (Maho et al., 2019; Olroyd & Sidor, 2017). The persistence and evolutionary stasis of *Mesenosaurus* implies a resilience across major ecological transitions and may be a result of the taxon occupying the mid‐sized carnivore niche in its the respective ecosystems, ecologically analogous to varanid lizards (Maho et al., 2019; Modesto et al., 2011; Williston, 1914). Relatively small varanid lizards, such as *V. niloticus*, are found in communities with diverse mammalian predators (Modesto et al., 2011; Pianka et al., 2004). Similarly, *M. efremovi* coexisted with a relatively larger sphenacodontid, *Dimetrodon* (Brink et al., 2019; Evans et al., 2009; Maho et al., 2024), temnospondyls, and a larger varanodontine varanopid at the Richards Spur locality. In *Mesenosaurus* and varanopids in general, the dentition is quite gracile, slender, and labiolingually compressed, more similar to extant varanids, whereas in sphenacodontids, with the exception of *Secodontosaurus* (Brink & Reisz, 2014), the dentition appears more bulbous at the base and are less subject to labiolingual stresses while feeding. Thus, their tooth morphology likely promoted powerful crushing and removing large chunks of prey in one piece. *Dimetrodon grandis* has large dentine cores, whereas *Dimetrodon limbatus* and *Secodontosaurus* do not have dentine‐cored serrations (Brink & Reisz, 2014). Therefore, the oldest appearance of true ziphodonty is found in *M. efremovi* and *D. grandis*, two carnivorous taxa which coexisted together. Based on its size, the large varanodontine varanopid at Richards Spur, *Varanops brevirostris*, may have likely been one of the apex predators in the ecosystem (Maddin et al., 2006; Maho et al., 2023); however, its dentition lacked the notable specializations associated with a hypercarnivorous diet, including serrated carinae. The possibility of a co‐occurring varanopid species and other large predators at Richards Spur implies that changes in the tooth morphology and dental specializations in *M. efremovi* may have been driven by competition.

## AUTHOR CONTRIBUTIONS

R.R.R. conceptualized the study. J.J.B., S.M., and T.M. performed the experiment. S.M. and T.M. analyzed the data, prepared figures and/or tables, and wrote the initial draft of the manuscript. All authors reviewed, edited, and approved the final draft of the manuscript.

## Supporting information


Data S1.

## Data Availability

The data that support the findings of this study are available from the corresponding author upon reasonable request.
