# Supplementary material for: Size and shape heterodonty in the early Permian synapsid Mesenosaurus efremovi
Source: J Anat. 2024 Mar 2;245(1):181–96. doi: 10.1111/joa.14034 (PMC11161827; doi:10.1111/joa.14034)
Supplement: Supplementary file 1 — Data S1. [file JOA-245-181-s001.pdf]

Supplementary Material

Size and shape heterodonty in the early Permian synapsid *Mesenosaurus efremovi*

Tea Maho[1,2], Sigi Maho[1], Joseph J. Bevitt[3], Robert R. Reisz[1,2]

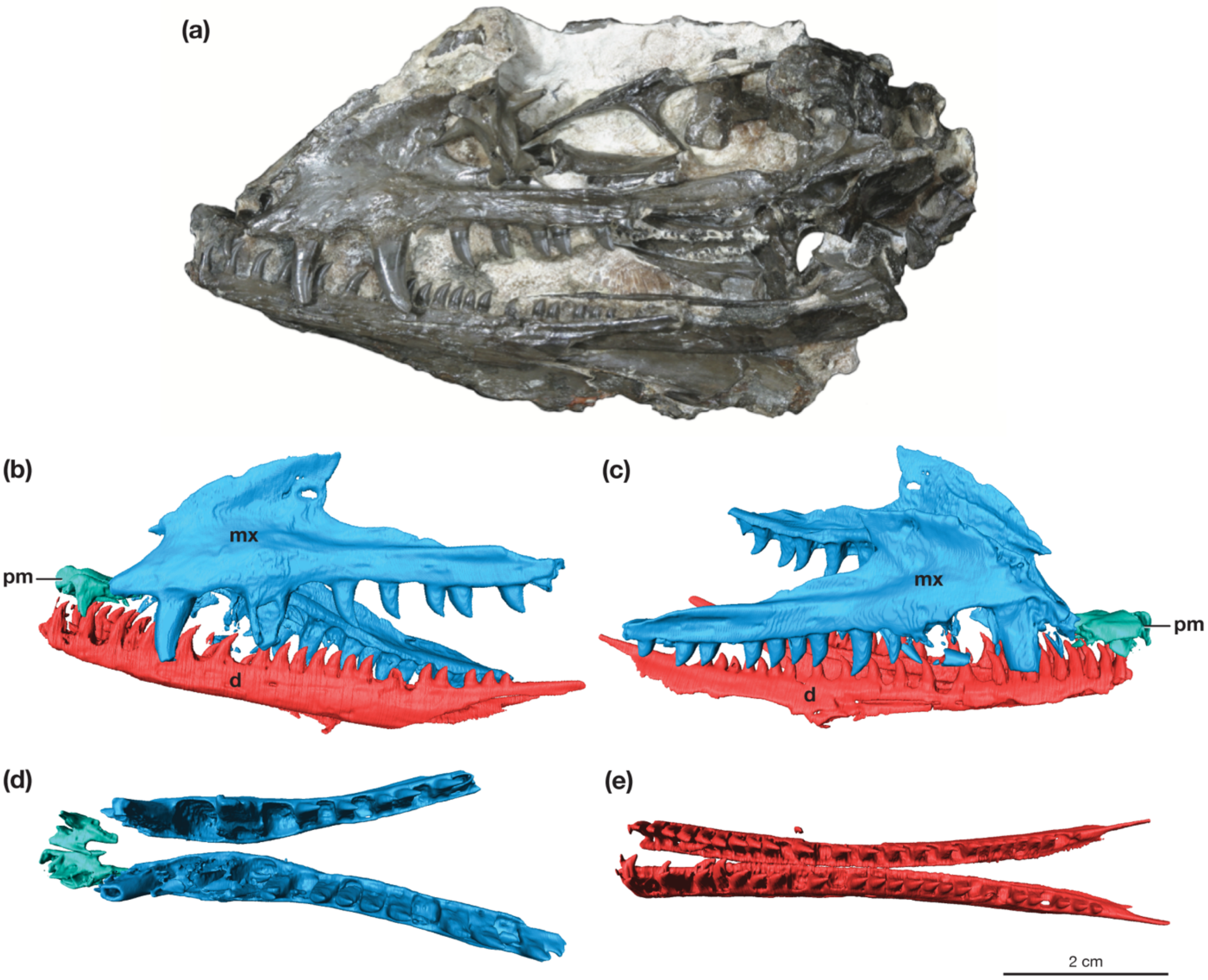

Figure S1. *Mesenosaurus efremovi,* holotype, OMNH 73209, with digital renderings of jawbones. (a) Photograph of left lateral view (Maho et al., 2019). Digital renderings of lateral views (b) left and (c) right. (d) Ventral/occlusal view of premaxillae and maxilla. (e) Dorsal/occlusal view of dentaries. Abbreviations: d, dentary; mx, maxilla; pm, premaxilla.

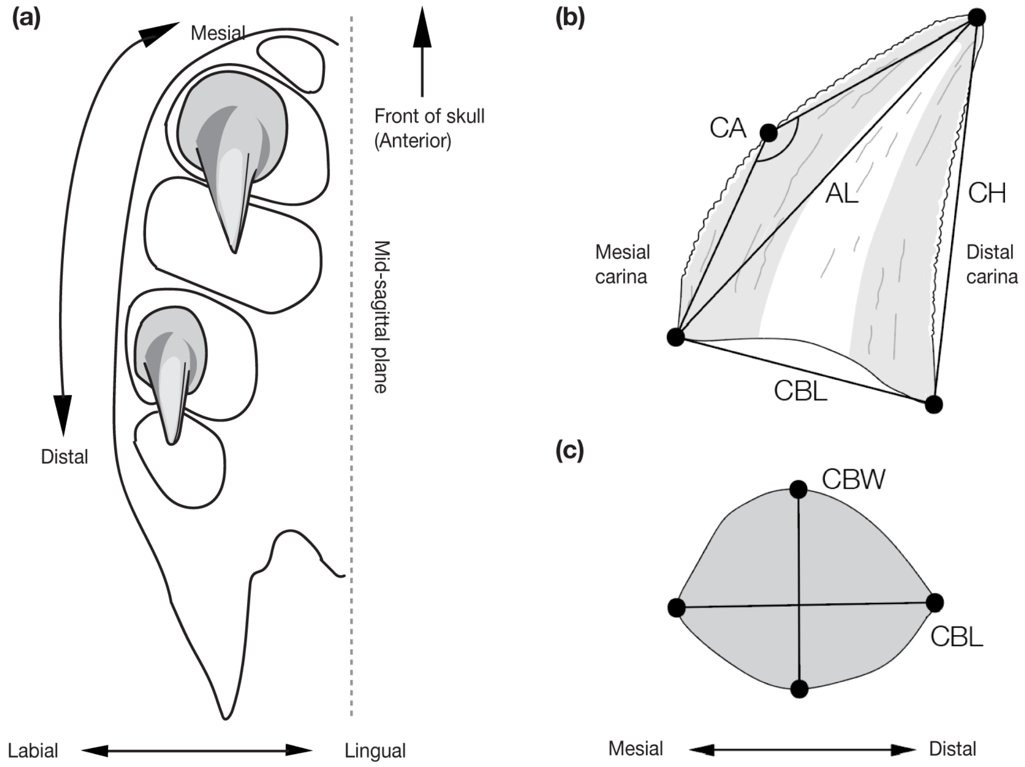

Figure S2. Anatomical orientations and variables measured on crowns of *Mesenosaurus efremovi* with respect to tooth position. (a) drawing of *Mesenosaurus efremovi* premaxilla, ROMVP 85458, in ventral view, showing mesiodistal and labiolingual orientations with respect to tooth crowns. (b) drawing of *Mesenosaurus efremovi* left dentary crown, OMNH 73209, in labial view, showing crown height, CH in mm; apical length, AL in mm; crown base length, CBL in mm; crown angle, CA in °, mesial carinae, and distal carina. (c) the crown in B in basal view showing CBL; and crown base width, CBW in mm. Figure concept after Smith (2005).

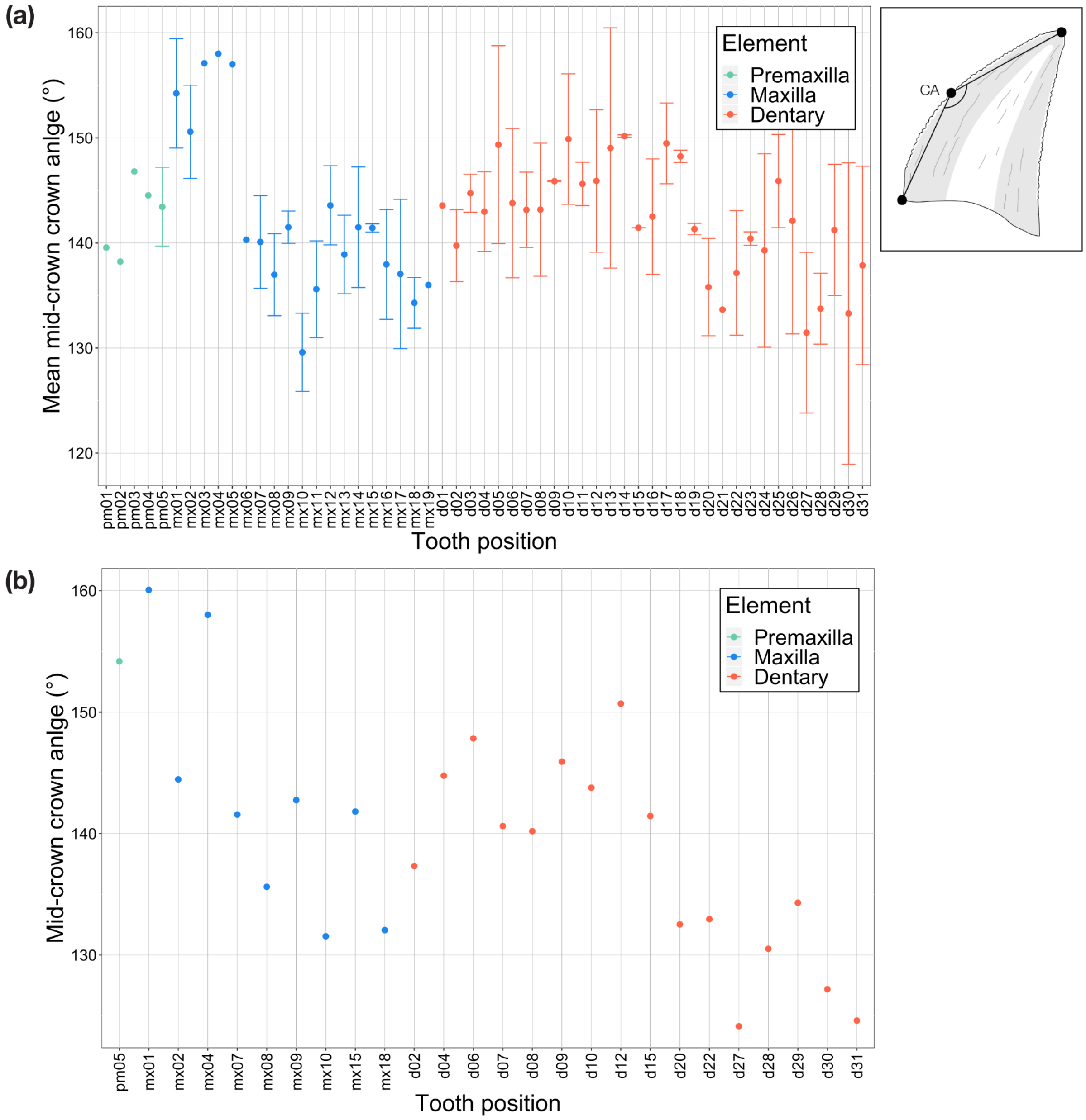

Figure S3. Crown angle (in °) with respect to tooth position for premaxillary, maxillary, and dentary dentition of *Mesenosaurus efremovi*. (a) mean mid-crown angle, n=41. (b) mid-crown angle of ROMVP 85440 marginal dentition, n=1. Green represents the premaxilla, blue represents the maxilla, and red represents the dentary.

Table S1. List of specimens and thin sections used in the study assigned to *Mesenosaurus efremovi*.

Specimen	Catalogue number	Specimen thin section number (TSN)
Skull	OMNH 73208	none
Skull	OMNH 73209	none
Skull	OMNH 73500	none
Snout	ROMVP 85439	none
Skull within a larger block of fragmentary material	ROMVP 85440	none
Skull	ROMVP 85441	none
Maxilla	ROMVP 85443	01610, 01612
Maxilla	ROMVP 85445	01613, 01614
Maxilla	ROMVP 85449	01634
Dentary	ROMVP 85453	01634, 01637
Maxilla	ROMVP 85455	01615, 01615
Maxilla	ROMVP 85456	none
Maxilla	ROMVP 85457	none
Premaxilla	ROMVP 85458	none
Premaxilla	ROMVP 85462	none
Premaxilla	ROMVP 85463	none
Premaxilla	ROMVP 85464	none
Premaxilla	ROMVP 85465	none
Premaxilla	ROMVP 85466	none
Premaxilla	ROMVP 85467	none
Premaxilla	ROMVP 85468	none
Maxilla	ROMVP 85469	none
Maxilla	ROMVP 85470	none
Maxilla	ROMVP 85471	none
Maxilla	ROMVP 85472	none
Maxilla	ROMVP 85473	none
Maxilla	ROMVP 85474	none
Maxilla	ROMVP 85475	none
Maxilla	ROMVP 85476	none
Maxilla	ROMVP 85477	none
Maxilla	ROMVP 85478	none
Maxilla	ROMVP 85479	none
Maxilla	ROMVP 85480	none
Maxilla	ROMVP 85481	none
Maxilla	ROMVP 85482	none

Maxilla	ROMVP 85483	none
Maxilla	ROMVP 85484	none
Maxilla	ROMVP 85485	none
Maxilla	ROMVP 85486	none
Maxilla	ROMVP 85487	none
Maxilla	ROMVP 85488	none
Maxilla	ROMVP 85489	none
Maxilla	ROMVP 85490	none
Maxilla	ROMVP 85491	none
Dentary	ROMVP 85492	none
Dentary	ROMVP 85493	none
Dentary	ROMVP 85494	none
Dentary	ROMVP 85495	none
Dentary	ROMVP 85496	none
Dentary	ROMVP 85497	none
Dentary	ROMVP 85498	none
Dentary	ROMVP 85499	none
Maxilla	ROMVP 85500	none
Dentary	ROMVP 85501	none

Table S2. ANOVA results (F values and p-values) for each of the crown morphology variables (CH, CBL, CBW, AL, CA). Significant differences (*p*-value < 0.05) are shown in bold. Dash represents a *p*-value > 0.1 for the backward selection model.

**Morphological Measurement**		**Premaxilla**		**Maxilla**		**Dentary**	
		**F value**	***p*-value**	**F value**	***p*-value**	**F value**	***p*-value**
**Crown height (CH)**	**Tooth Position**	0.168	0.699	1.563	0.217	61.290	**7.11e-11**
	**Specimen**	3.904	0.095	2.905	**0.006**	38.076	**< 2.2e-16**
	**Position^2**	0.610	0.470	4.577	**0.037**	125.331	**< 2.2e-16**
	**Position:Specimen**	–	–	–	–	6.064	**8.99e-06**
	**Position^2:Specimen**	–	–	–	–	3.940	**0.0008**
**Crown base length (CBL)**	**Tooth Position**	1.112	0.339	18.26	**0.00017**	157.197	**< 2.2e-16**
	**Specimen**	2.569	0.171	4.424	**0.006**	20.884	**6.98e-16**
	**Position^2**	1.596	0.262	14.94	**0.00053**	89.539	**3.30e-14**
	**Position:Specimen**	–	–	–	–	3.338	**0.0027**
	**Position^2:Specimen**	–	–	–	–	–	–
**Crown base width (CBW)**	**Tooth Position**	1.113	0.351	0.4842	0.49649	12.591	**0.0008916**
	**Specimen**	1.693	0.263	15.849	**0.00016**	19 8199	**4.54e-12**
	**Position^2**	1.659	0.267	1.2844	0.27377	7.920	**7.12e-03**
	**Position:Specimen**	–	–	–	–	3.935	**0.0019**
	**Position^2:Specimen**	–	–	–	–	–	–
**Apical length (AL)**	**Tooth Position**	0.099	0.765	8.749	**0.00483**	88.522	**1.28e-13**
	**Specimen**	4.273	0.083	2.223	0.02885	30.614	**<2.2e-16**
	**Position^2**	0.334	0.588	12.56	0.0009	155.536	**<2.2e-16**
	**Position:Specimen**	–	–	–	–	4.972	**8.50e-05**
	**Position^2:Specimen**	–	–	–	–	2.691	**0.0130**
**Crown angle (CA)**	**Tooth Position**	1.658	0.327	41.22	**1.50e-06**	0.118	**0.7329**
	**Specimen**	N/A		2.973	0.01325	10.2361	10.236
	**Position^2**	1.347	0.366	26.63	**3.13e-05**	3.506	**0.0678**
	**Position:Specimen**	–	–	–	–	6.930	**0.0024**
	**Position^2:Specimen**	–	–	–	–	–	–

Table S3. Premaxillary, maxillary, and dentary tooth counts of *Mesenosaurus efremovi* specimens.

Element	Specimen Number	Element	Side	Positions	Length (mm)
**Premaxilla**	OMNH 73500	Premaxilla	Right	5	11
**Premaxilla**	OMNH 73208	Premaxilla	Right	5	8
**Premaxilla**	ROMVP 85439	Premaxilla	Right	5	9
**Premaxilla**	ROMVP 85439	Premaxilla	Left	5	9
**Premaxilla**	ROMVP 85440	Premaxilla	Left	5	13
**Maxilla**	OMNH 73500	Maxilla	Right	19	58
**Maxilla**	OMNH 73500	Maxilla	Left	19	58
**Maxilla**	ROMVP 85441	Maxilla	Right	21	49
**Maxilla**	ROMVP 85440	Maxilla	Left	19	58
**Dentary**	ROMVP 85440	Dentary	Right	30/31	88
**Dentary**	OMNH 73208	Dentary	Right	31	55
**Dentary**	OMNH 73209	Dentary	Right	31	65
